# 41 Twenty years’ experience with juvenile dermatomyositis among Libyan children

**DOI:** 10.1093/rheumatology/keac496.037

**Published:** 2022-10-07

**Authors:** Soad Hashad, Hala Etayari, Awatif Abushhaiwia, Majeda Eltofil, Aya Twati, Zuhaira Awhidah, Aeshah Ateeq, Yusra Elfawires

**Affiliations:** 1 Tripoli Children’s Hospital; 2 The University of Tripoli

## Abstract

**Background:**

Juvenile inflammatory myositis are systemic autoimmune diseases of unknown aetiology that are characterized by inflammation of skeleton, muscles, skin, and internal organs. Studies from different regions have reported different incidence and age at onset suggesting that the clinical and demographic features may differ by race and geographic regions. This study aims to describe the characteristics of disease among Libyan children who have been treated in the main rheumatology unit covering most of the population in Libya

**Objectives:**

To describe the demographic and clinical features of patients with juvenile dermatomyositis

To determine the outcome of children with juvenile dermatomyositis and factors affecting the outcome.

**Patients and methods:**

This is a retrospective descriptive study conducted by reviewing patient records diagnosed with juvenile dermatomyositis from 5/2000–2/2022.

**Results:**

Twenty-one patients were included, female to male ratio was 6:1. Mean age at disease onset was 7.7 ± 2.8 years and their mean follow-up period was 4.36 ± 3.2 years. Most of the patients (14.7%) presented before 1 month of starting symptoms. Most of the patients (13,0.9%), were diagnosed as dermatomyositis, 2 (9.5%) as polymyositis, 2 (9.5%) as amyopathic myositis, and 4 (19%) as overlap syndrome. Family History of dermatomyositis was positive in 4 (19%) of patients and family history of other autoimmune diseases was positive in 5 (23.8%) patients.

Around half of the patients had monocyclic disease course (11, 52.4%), 5 patients (23.8%) had polycyclic disease course and other 5 patients (23.8%) had chronic persistent disease course.

MRI proximal muscles and EMG were used for diagnosis in 10 patients (48%) and 16 (76.1%) respectively, and muscle biopsy was used in 2 patients to confirm the diagnosis.

One overlap patient had severe lung fibrosis on CT scan chest with restrictive lung disease and 4 patients (19%) had restrictive lung disease with normal CT scan chest.

Methyleprednisolone pulses were used in 10 patients (50%) with 3 (15%) who required more than one pulse of intravenous prednisolone. Immunoglobulin was used in 12 (60%) of the patients. All patients needed oral prednisolone and methotrexate was used in 15 (75%) of the patients. Other drugs used were Azathioprine in 3 overlap patients, cyclophosphamide and Mycophenolate mofetil in one patient with overlap syndrome. Hydroxychroroquine was used in 6 (30%) of the patients.

In the last visit, 12 patients (60%) were in remission, 7 (58%) of them presented before 1 month of disease onset. Fifty-eight percent of the females and 66.7% of the males were in remission. Five patients (25%) had short stature, 3 (15%) had chronic cutaneous changes, 1 (5%) had arrythmia, 2 (10%) had calcinosis, 2 (10%) had osteoporosis, and one patient died.

**Conclusion:**

A female predominance was noted with age at presentation comparable to other studies.

The most frequent manifestations were skin manifestations and musculoskeletal features were the second most frequent symptoms.

Patients has low rate of complications with low rate of calcinosis and mortality and none of them had gastrointestinal manifestations.

Males were more likely to be in remission.

**41 Table 1:** summarizes the main clinical features at presentation:

Feature	Number and percentage
Skin changes	17 (81%)
Facial oedema	1 (4.8%)
Hands and feet oedema	1 (4.8%)
Fever	4 (19%)
Generalized fatigability	12 (57.1%)
Arthritis	11 (52.4%)
Arthralgia	1 (4.8%)
Muscle weakness	15 (71%)
Dysphonia	4 (19%)
Contractures	2 (9.5%)

